# LSD1: more than demethylation of histone lysine residues

**DOI:** 10.1038/s12276-020-00542-2

**Published:** 2020-12-14

**Authors:** Bruno Perillo, Alfonso Tramontano, Antonio Pezone, Antimo Migliaccio

**Affiliations:** 1 Istituto per l’Endocrinologia e l’Oncologia Sperimentale “G. Salvatore” C.N.R, 80131 Naples, Italy; 2 Dipartimento di Medicina di Precisione Università della Campania “L. Vanvitelli”, 80138 Naples, Italy; 3 Dipartimento di Medicina Molecolare e Biotecnologie Mediche Università Federico II, 80131 Naples, Italy

**Keywords:** Epigenetics, Histone post-translational modifications

## Abstract

Lysine-specific histone demethylase 1 (LSD1) represents the first example of an identified nuclear protein with histone demethylase activity. In particular, it plays a special role in the epigenetic regulation of gene expression, as it removes methyl groups from mono- and dimethylated lysine 4 and/or lysine 9 on histone H3 (H3K4me1/2 and H3K9me1/2), behaving as a repressor or activator of gene expression, respectively. Moreover, it has been recently found to demethylate monomethylated and dimethylated lysine 20 in histone H4 and to contribute to the balance of several other methylated lysine residues in histone H3 (i.e., H3K27, H3K36, and H3K79). Furthermore, in recent years, a plethora of nonhistone proteins have been detected as targets of LSD1 activity, suggesting that this demethylase is a fundamental player in the regulation of multiple pathways triggered in several cellular processes, including cancer progression. In this review, we analyze the molecular mechanism by which LSD1 displays its dual effect on gene expression (related to the specific lysine target), placing final emphasis on the use of pharmacological inhibitors of its activity in future clinical studies to fight cancer.

## Cancer: Enzyme modifying chromosomal proteins needs closer examination

Further research into the complex structure and behavior of an enzyme involved in gene regulation could improve future cancer therapies. The modification of chromosomal proteins known as histones can fundamentally change gene expression and influence the progression of diseases such as cancer. Bruno Perillo at the Italian National Research Council, Naples, Italy, and co-workers reviewed understanding of the structurally complex enzyme lysine-specific histone demethylase 1 A (LSD1), which interacts with multiple targets including histones. LSD1 removes methyl groups from histones, fine-tuning gene expression and influencing protein activity. The overexpression of LSD1 is linked to cancer development, particularly in aggressive cancers, and inhibiting LSD1 has shown promise in slowing progression and cancer spread. The researchers call for further research into the complexities of LSD1 activity, both in cancers and normal cell function.

## Introduction

Nucleosomal histones (H2A, H2B, H3, and H4) are extensively involved in DNA supercoiling and chromosomal positioning within the nuclear space. Multiple biochemical groups (with acetylation, methylation, and phosphorylation being the most frequent) can be added to specific amino acids in the N-terminal tails of histones, and these diverse posttranslational modifications (PTMs), deciphered in the histone code^1,2^, control the dynamic plasticity of chromatin, allowing beneficial interactions with nuclear protein complexes that govern gene expression, and DNA replication, repair and recombination^3^. In contrast to acetylation, which was previously recognized as a dynamic process generated by activity of histone acetyltransferases (HATs) and histone deacetylases (HDACs)^4^, methylation of histones was considered an irreversible process for a long time. However, almost two decades ago, histone demethylating activity was detected in chromatin corepressor complexes and was attributed to lysine-specific histone demethylase 1 (LSD1), which removes monomethyl and dimethyl groups from lysine 4 in histone H3 (H3K4me1/2), acting as a repressor of gene expression^5,6^. While acetylation and, in most cases, phosphorylation are associated mainly with transcription activation, methylation is considered an inhibitory or an activating mark, with H3K4 methylation triggering transcriptional stimulation^7^ and H3 lysine 9 methylation triggering transcriptional repression^8^.

## LSD1 structure: an overview

**Fig. 1:**
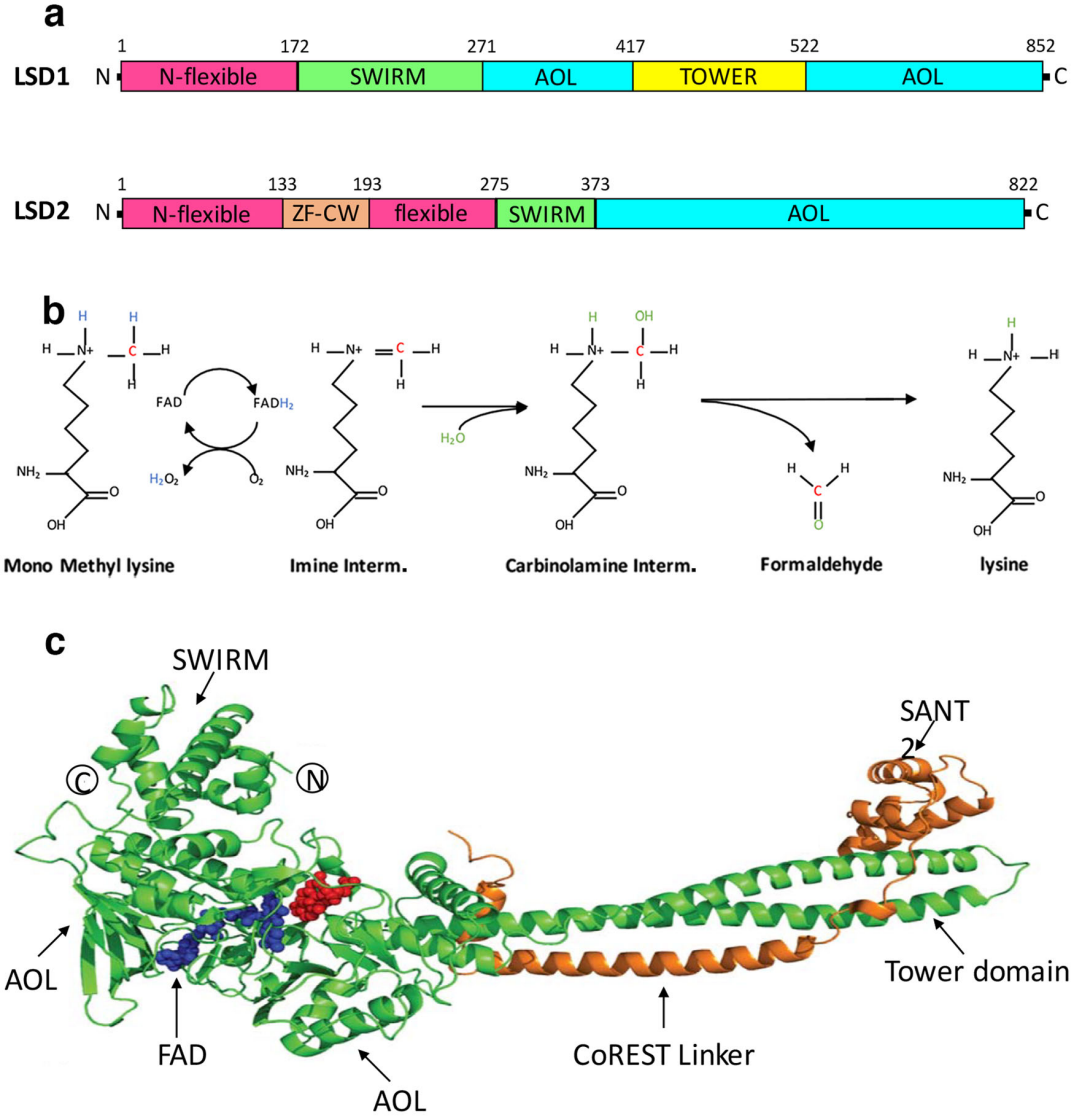
Schematic representation of LSD1 and LSD2 class I demethylases. **a** Cognate domains in each enzyme are reported with identical colors to highlight the similarities. **b** The pathway of lysine demethylation shows the formation of H_2_O_2_ and formaldehyde as side products and the imine intermediate that requires protonated lysine and accounts for the inability of LSD1 to demethylate trimethyl residues. **c** Graphic representation of the LSD1 interaction with the SANT2 domain of CoREST (adapted from ref. ^25^).

LSD1, also known as KDM1A (lysine-specific demethylase 1A) or AOF2 (flavin-containing amine oxidase domain-containing protein 2), is an 852 amino acid flavin-dependent monoamine oxidase (MAO) protein of 110 kDa. LSD1 and its homolog, LSD2 (or KDM1B, AOF1), were the first recognized members of the FAD-dependent family of protein demethylases^9^. As FAD-dependent enzymes, LSD1 and LSD2 belong in the class I demethylase category^10^, with LSD2 showing approximately 30% sequence homology and similar structural properties with its sister demethylase^11^. LSD1 consists of three different domains, as defined by crystallography studies: the Swi3/Rcs8/Moira (SWIRM, aa 172–270) domain, the Tower domain (aa 417–521), and the catalytic amine oxidase-like (AOL) domain (aa 271–416 and 522–852) (Fig. 1a). The SWIRM domain, located immediately downstream from the N-terminal portion that is important for nuclear localization (aa 1–171)^12^, is a helical region that folds back against the catalytic C-terminal site, contributing to the formation of a groove that is involved in substrate binding^13,14^. The C-terminal domain is split into two fragments by the Tower domain: the left side essentially binds the flavin adenine dinucleotide (FAD) cofactor (even though it cooperatively functions with specific fragments of the right side, aa 559–657 and 770–833), while the right side interacts with SWIRM and forms a cavity that represents the catalytic pocket of LSD1 (aa 523–558 and 658–769, in association with aa 357–416 of the left side). The Tower domain protrudes from this spherical protein core and forms a slim helix-turn-helix motif that provides the binding site for the SWI3/ADA2/N-CoR/TFIIIB (SANT) 2 domain in the corepressor for element-1-silencing transcription factor (CoREST) complex that, in turn, facilitates LSD1 tethering to the nucleosomal substrate^15^, which is wedged by the Tower domain itself^16^. When LSD1 binds a monomethylated or dimethylated lysine, FAD oxidizes the alpha carbon of the methyl group, generating an imine intermediate and FADH_2_, which is then reoxidized to FAD by molecular oxygen, driving the formation of hydrogen peroxide (H_2_O_2_)^6^.

Although large, the LSD1 catalytic pocket is not deep enough to accommodate substrates with more than three residues on the N-terminus side of the methylated lysine; LSD1 as a demethylase preferentially removes methyl groups from monomethylated and dimethylated lysine 4 in histone H3^17^. The inability of LSD1 to remove trimethyl groups is explained by the need for protonated lysine residues throughout the reaction, and trimethyl-lysine residues are not protonated (Fig. 1b)^18^. In contrast to LSD1, its sister demethylase, LSD2, does not have a Tower domain but has a zinc-finger cysteine-tryptophan (ZF-CW) domain that, in this case, interacts with N-terminal SWIRM, which mediates its demethylase activity^19^.

### LSD1 as an epigenetic driver of transcriptional repression

Transcriptional repression induced by LSD1 upon removal of methyl groups from H3K4me1/2, was initially linked via its association with the transcriptional corepressor protein complex CoREST^20^. The human CoREST family is formed by three proteins that are encoded by separate genes (*CoREST1*, *CoREST2*, and *CoREST3*) whose founding member, CoREST1, was first characterized as a partner of the RE1-silencing transcription factor/neural-restrictive silencing factor (REST/NRSF)^21^, which is crucial for the control of neuronal gene expression^22,23^. CoREST proteins do not have signature domains but contain two SANT domains, one of which (SANT2) gives LSD1 the ability to demethylate lysine residues present within the nucleosome^24,25^ (Fig. 1c). CoREST complexes also enclose histone deacetylases 1/2 (HDAC1/2) and the plant homeodomain (PHD) finger protein BHC80: in this way, they combine two transcriptional inhibitory factors (LSD1 and HDAC1/2), with LSD1 activity, which is positively influenced by HDAC-induced deacetylation that increases affinity of the entire complex for chromatin, promoting the remove of methyl marks from target lysine residues by demethylase^26,27^. Interestingly, even though LSD1 is present in all three members of the CoREST family, its association with CoREST3 results in an antagonistic effect on the demethylase activity shown by CoREST1 complexes, suggesting that LSD1 function varies upon interchanges with different members of the CoREST family, depicting different scenarios for cell differentiation^28^.

LSD1 also interacts with Snail/Gfi-1 (SNAG) family zinc finger proteins that repress transcription^29^. Specifically, since the SNAG domain appears to be a histone H3-mimicking motif that shows high affinity for the AOL domain of LSD1, it behaves as a molecular hook between a demethylase and its cofactors^30^, similar to the genes involved in the control of hematopoietic differentiation^31^ or metastatic diffusion in acute myeloid leukemia^32^, either in a lineage-specific manner^33^ or not^34^. LSD1, in fact, plays a relevant role in acute myeloid leukemia (AML), where it behaves as a modulator of hematopoiesis and leukemogenesis by maintaining stem cell self-renewal and regulating cell differentiation in hematopoietic stem cells (HSCs) and early myeloblasts^35,36^, by interacting with transcription factors and chromatin-modifying enzymes^37,38^. LSD1 is also involved in the regulation of the progression of AML. It acts as an essential modulator of leukemia stem cell (LSC) differentiation^39^, where sustained expression of the fusion gene methyltransferase mixed lineage leukemia (MLL)-AF9 is necessary for the cycle progression of LSCs.

However, connections between different families of protein complexes that were once viewed as distinct from one another are supported by growing evidence, that reveals a picture of dynamic intersected assemblies that regulate essential cellular processes. In fact, LSD1 is also a component of the Mi-2/nucleosome remodeling deacetylase (NuRD) complex, a multiprotein complex that combines functions including, among others, the deacetylase activity of HDAC1/2 with the nucleosome remodeling of the ATPases chromo domain helicase DNA-binding protein (CHD) 3 and CHD4 and the methyl-CpG-binding domain proteins MBD2 or MBD3, thus playing pivotal roles in the DNA damage repair process^40^ and in the generation of a repressive chromatin state during development and differentiation^41^. The association of LSD1 with NuRD complexes has been recently found to suppress breast cancer metastasis and to decommission genes involved in pluripotency programs in embryonic stem cells (EMCs), which is important for the transition of these cells to other (more specialized) states^42,43^. LSD1 also controls cell growth and chemoresistance by coordinating with the SIN3A/HDAC complex, providing the basis for the interplay between factors that control histone methylation and acetylation in the expression programs of oncogenes (repressed) while ensuring breast cancer growth^44,45^. Moreover, it reduces the levels of the tumor suppressor Lefty1, which is overexpressed in ovarian clear cell carcinomas through its interaction with β-catenin^46^ and is characterized as a double-edged sword in the control of cancer cell survival.

Finally, LSD1 behaves as a complex histone modifier in the maintenance of cellular pluripotency, as it regulates the correct balance of two opposite marks in poised genes, the repressive demethylation of H3K4 (LSD1-dependent) and the stimulatory demethylation of H3K27^33,47^, and the selective knockdown of LSD1 resulted in an increase in methylated lysine 36 and 79 in histone H3, suggesting that LSD1 collaborates with other demethylases in the regulation of gene expression^48^.

### LSD1 as an epigenetic driver of transcriptional stimulation

**Fig. 2:**
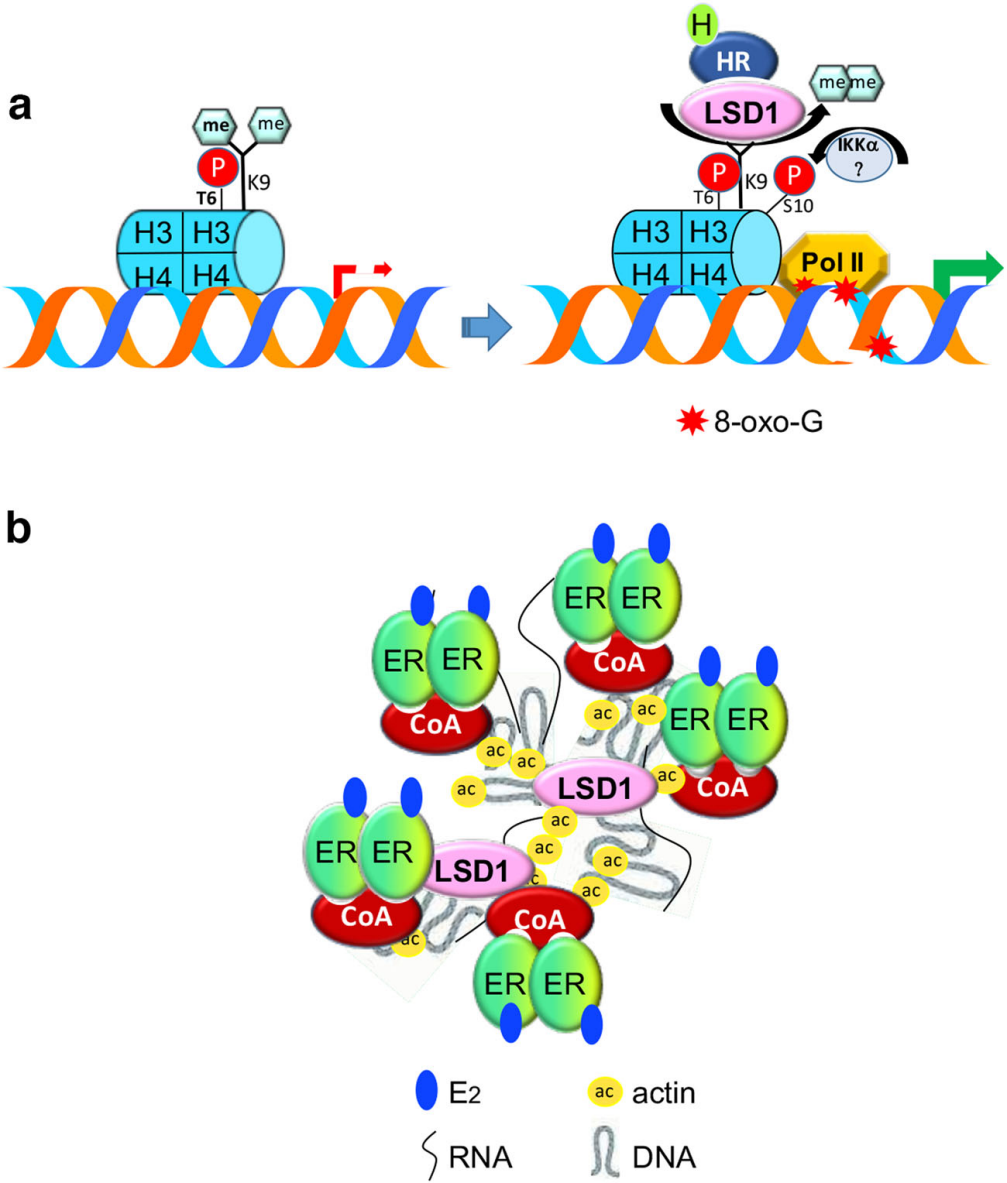
LSD1 interactions with nuclear receptors. **a** In the absence of hormones, LSD1 is absent in chromatin or, in the case of progesterone, is sequestered with unliganded receptors (PRs) at the responsive sites of target genes assembled within the CoREST complex (not shown). Under these conditions, the relevant nucleosomes are devoid of methylated lysine 4. After hormone challenge, LSD1 interacts with liganded receptors and targets lysine 9 in histone H3 to stimulate the transcription of responsive genes. The shift is driven by phosphorylation of the nearby threonine 6 within the same histone and induced by hormone. This demethylation also generates ROS (H_2_O_2_) that induce the oxidation of nearby guanines with consequent repair and nicks on DNA to govern chromatin plasticity and the subsequent assembly of the transcription machinery. H3K9 remains demethylated when the adjacent H3S10 is phosphorylated by kinases, including the NFkB inducer IkB kinase α (IKKα). **b** Role of LSD1 in the formation of transcription factories, sites of concomitant transcription of multiple estrogen-responsive genes from different chromosomes, assembled at specific loci by mediation of actin granules hooked by the demethylase.

Interestingly, LSD1 also interacts on chromatin with nuclear receptors, but in this case, it behaves as an inducer of gene expression. Specifically, LSD1 binds the androgen receptor and promotes androgen-dependent transcription of hormone-responsive genes, enhancing tumor-cell growth: this action is due to a change in its target, the transcription repressive mark monomethyl and dimethyl-lysine 9 in histone H3^49,50^. The switch of LSD1 enzymatic specificity with the subsequent driving of transcription complex recruitment is due to the phosphorylation of threonine 6 in histone H3 (H3T6), which is triggered by protein kinase C β (PKCβ) and is activated by hormones^51^, enabling the interaction of H3K9me1/2 with the LSD1 catalytic pocket (Fig. 2a). In this regard, since LSD1 is able to remove exclusively monomethyl and dimethyl groups from target lysine residues, to obtain complete H3K9 demethylation, a member of the Jumonji C (JmjC) domain-containing family of demethylases that depend on Fe(II) and α-ketoglutarate as cofactors and produce free lysine and formaldehyde as final products, joins the complex^52^. In androgen-responsive cells, LSD1 collaborates with the Jumonji domain-containing JMJD2C demethylase to stimulate the expression of androgen-target genes^53^.

LSD1, in association with the JMJD2A demethylase, is also involved in transcriptional activation of estrogen-responsive genes, with subsequent stimulation of cell proliferation and tumor growth: its recruitment to promoter/enhancers increases after hormone treatment and interaction with a liganded receptor^54,55^. The effect of LSD1 on transcriptional regulation is mediated by the H_2_O_2_ generated by the reoxidation of FADH_2_, which induces oxidation of guanines (Gs) on nearby DNA, triggering the activation of the base excision repair (BER) enzymatic system with the formation of nicks and regulation of chromatin plasticity to allow bridging between the regulatory 5′ and the 3′ ends of inducible genes^56,57^ (Fig. 2a). Importantly, excess oxygen reactive species (ROS) are first subjected to a fine-tuning control mechanism^58^, and then scavenged by nuclear superoxide dismutase (SOD) enzymes^59,60^. LSD1 is closely correlated with the control of cell proliferation, angiogenesis, migration, and invasion in breast cancer^61^ and prostate cancer^62,63^, where it contributes strongly to carcinogenesis induced by sex hormones^53,64^, either by inducing DNA oxidation and transcription of cell cycle regulatory genes^56^ or by modulating the expression of AR-independent or AR-dependent survival genes in castration-resistant prostate cancer (*CRPC*) cells, where it is overexpressed^65–67^.

The use of LSD1-generated ROS to trigger gene expression raises several questions. What happens when the demethylase inhibits transcription as a consequence of its removal of methyl marks from H3K4? Does it generate H_2_O_2_ in this case? If it does, as can be presumed, what are the roles of ROS, if any? These questions still await answers. Moreover, the use of transcription-generated ROS may be useful in anticancer therapies. In fact, based on their potential dangerous effect on DNA stability, ROS production can be induced by disrupting finely controlled mechanisms until they reach levels at which cells trigger the apoptosis program, with obvious positive outcomes against cancerous cells^55^.

An original role has been recently proposed for LSD1 in the control of the coordinated expression of genes responsive to estrogens. Multiple genes sensitive to a particular stimulus are simultaneously expressed at discrete sites within the nucleus, termed transcription factories, where multiple RNA polymerases are anchored to subnuclear structures that are critical for interchromosomal interactions and are named interchromatin granules, formed essentially by actin^68–71^. Here, LSD1 functions as a hook that connects multiple liganded estrogen receptor molecules recruited to different regulatory sites of target genes through interactions with the assembled cofactors and nuclear actin granules^72^ (Fig. 2b).

LSD1 also plays a pivotal role in transcriptional activation and chromatin loop establishment of retinoic acid-responsive genes^73^. In fact, a complex cross-talk links the estrogen receptor (ERα) with the retinoic acid receptor (RARα), which has an opposite effect in most cell lines. Gene expression analysis has revealed that either transcription of RARα is induced by estrogens and that ERα expression is induced by retinoic acid (RA)^74,75^. Moreover, while the two receptors share a subset of binding regions on chromatin, suggesting that they compete for binding to the same sites^71^, RARα cooperates with ERα on estrogen (E2)-responsive loci to drive the expression of E2 target genes^76^.

Finally, a peculiar mechanism for LSD1 action on hormone-inducible genes has been recently highlighted for progesterone. In contrast to what has been shown with cognate nuclear receptors (i.e., ERα, RARα and AR), where LSD1 interacts with liganded receptors and is recruited to promoters/enhancers of target genes^77^, LSD1 assembles with free progesterone receptors (PRs) to inhibit the transcription of progesterone-inducible genes by maintaining the CoREST complex on the promoter of the responsive genes^78^. When cells are challenged with the hormone, LSD1 loses multiprotein assembly and drives the release of the entire repressor complex from chromatin, freeing downstream genes for productive transcription^78^.

### LSD1 as demethylase of nonhistone proteins

**Fig. 3:**
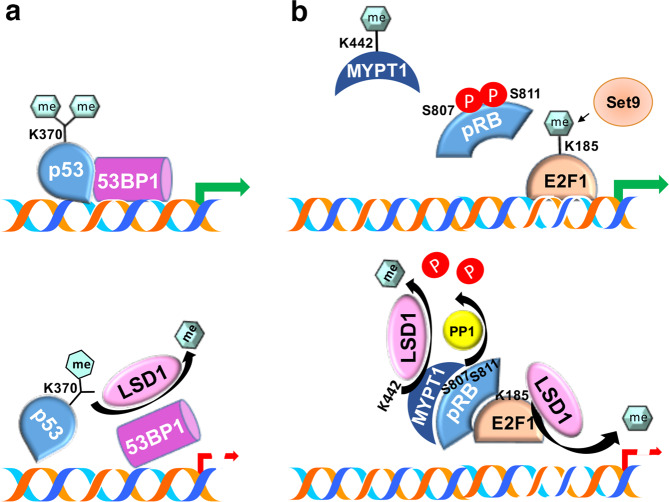
LSD1 interactions with nonhistone proteins (I). **a** p53, the pivotal regulator of multiple processes of cellular life (i.e., cell cycle progression, genomic stability and programmed cell death), when dimethylated at lysine 370, assembles with its cofactor 53BP1 and targets chromatin to activate transcription. If LSD1 removes one methyl group from the same lysine, this interaction is abrogated, and the expression of p53 target genes is inhibited. **b** pRB, the first identified tumor suppressor protein, with the two serine residues phosphorylated as shown is unable to heterodimerize with the E2F1 transcription factor. Both phosphate groups can be removed by the MYPT1 phosphatase, enabling the protein to interact with E2F1. The activity of MYPT1 is, in turn, dependent on the methylation status of lysine 442, which is under the control of LSD1.

LSD1 is able to demethylate nonhistone proteins: the tumor suppressor protein p53 is the first nonhistone protein identified as an LSD1 substrate in CRPC cells^79^. p53, which controls progression of the cell cycle, programmed cell death, genomic stability, and maintenance of stemness^80^, shows multiple methylated lysine residues, among which K370 plays a relevant role, with each level of its methylation displaying different biological effects^81^: when K370 is dimethylated, p53 binds its cofactor p53-binding protein 1 (53BP1) and activates p53 target genes; if, on the other hand, LSD1 removes one methyl group from K370, p53 loses its stimulatory effect^81^ (Fig. 3a).

Interestingly, LSD1 also affects the activity of the first identified tumor suppressor protein, the product of the retinoblastoma gene (*RB1*), which leads to arrest in the G_1_ phase of the cell cycle^82^. pRB acts in concert with another key regulator of cell cycle progression, the transcription factor E2F1. Together, these factors control cell cycle progression through the G_1_/S transition and the induction of DNA repair and programmed cell death. pRB activation is controlled by the phosphorylation of its serine 807 and 811, mediated by cyclin-dependent kinases (CDKs), inhibiting heterodimerization with E2F1 and allowing it to activate the expression of target genes; on the other hand, pRB dephosphorylation is mediated by myosin phosphatase target subunit 1 (MYPT1). MYPT1 is demethylated, in turn, at lysine 442 by LSD1^83^, with consequences for pRB/E2F1 heterodimerization^84^ (Fig. 3b). However, LSD1 also stabilizes E2F1 through the demethylation of lysine 185, previously methylated by the suppressor of variation, enhancer of zeste and trithorax 9 (Set9) methyltransferase, a methylation modification that makes the transcription factor prone to ubiquitination^85^ (Fig. 3b).

Methylation of histones is functionally linked to DNA methylation, which is, in turn, inversely involved in transcriptional induction^86–88^. This pattern is controlled by LSD1 throughout gastrulation in embryogenesis, affecting the stability of the DNA methyltransferase 1 (DNMT1) protein. DNMT1 is methylated on several lysine residues by Set7/9 methyltransferase, a process that enhances DNMT1 degradation; on the other hand, LSD1-dependent demethylation stabilizes DNMT1^89^.

LSD1 also controls the turnover of hypoxia-inducible factor 1-alpha (HIF-1α), the master transcriptional regulator of the cellular response to hypoxia^90^. It inhibits HIF-1α downregulation by demethylating the K391 residue, while HIF-1 α side product, H_2_O_2_, inhibits the hydroxylating activity of prolyl hydroxylase domain protein 2 (PHD_2_) on HIF-1α with its subsequent ubiquitination, thus promoting protein stability and tumor angiogenesis^91^.

**Fig. 4:**
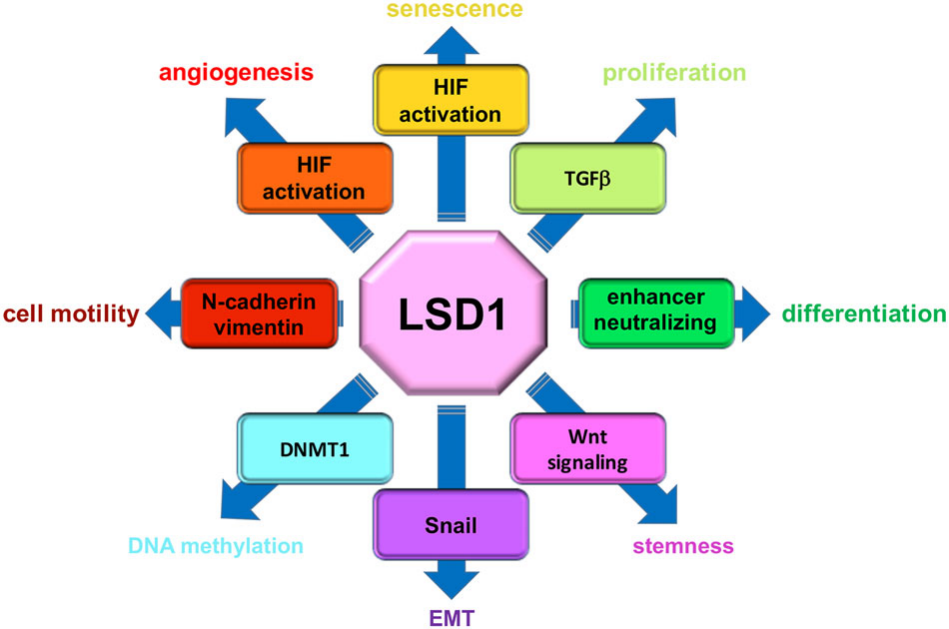
LSD1 interactions with nonhistone proteins (II). LSD1 is a central regulator of multiple relevant processes of cellular life, important either for general homeostasis or (if deregulated) tumor progression. The graphical scheme shows the target proteins that mediate the multifaceted effect of LSD1 activity on cellular life.

It is beyond the scope of this review to list in detail all the nonhistone proteins whose activity is influenced by LSD1. We emphasize that LSD1, by controlling the methylation status of either histone or nonhistone proteins, is extensively involved in the control of several cellular processes in multiple cellular and tissue environments, including cell proliferation^92–94^, differentiation^95,96^, and stemness via the activation of the β-catenin signaling^97,98^; the chemoresistance of several cancers, in which it appears to be upregulated^99^, the epithelial–mesenchymal transition (EMT)^100,101^, DNA methylation (with subsequent metastasis)^89,102^, cell motility^103,104^, angiogenesis^91^, and senescence^105^ (Fig. 4).

### LSD1 as target of post-translational modification

**Fig. 5:**
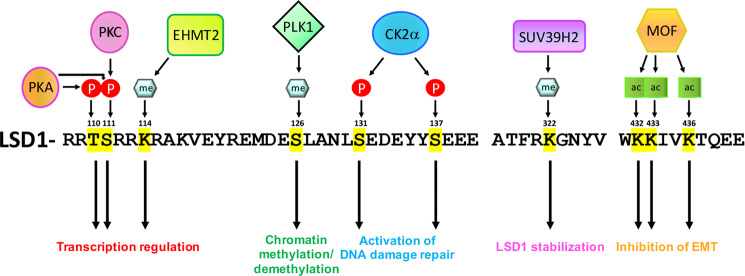
Multiple sites of LSD1 post-translational modifications. The most relevant processes under the respective control and the proteins critical for each PTM have been reported.

LSD1 is itself subjected to post-translational modifications^106^. It is, in fact, methylated at lysine 114 by euchromatic histone-lysine N-methyltransferase 2 (EHMT2) after androgen stimulation, with the subsequent recruitment of chromodomain helicase DNA-binding protein 1 (CHD1), which favors transcription^107,108^ (Fig. 5).

LSD1 can also be phosphorylated by protein kinase A (PKA) on threonine 110 and/or serine 111, a modification required for the recruitment of CoREST and HDAC complexes to estrogen-responsive genes^109^ (Fig. 5). The latter residue can also be phosphorylated by protein kinase C (PKC) in different pathways (Fig. 5): LSD1 phosphorylation on S111 is required for the activation of the inflammatory response because it regulates the stability of p65, a component of the nuclear factor kappa-light-chain-enhancer of activated B cells (NF-κB) complex that controls the transcription of genes necessary to amplify the inflammatory response^110^. The phosphorylation of serine 111 by PKCα and PKCθ also enhances the binding of HDAC, with the subsequent regulation of presynaptic genes and hippocampus-dependent memory^111^ or the control of circadian rhythmicity^112^. In this regard, phosphorylation of S111 is required to induce the formation of a complex with circadian locomotor output cycles kaput (CLOCK) and brain and muscle Arnt-like protein-1 (BMAIL1) (the master genes that drive rhythmic gene expression) to facilitate E-box-mediated transcriptional activation^113^. The phosphorylation of S111 is also important to the role that LSD1-induced transcriptional activity plays in the induction of the EMT and metastasis in breast cancer cells^114,115^. Another PTM important in this regard is its acetylation on lysine 432, 433, and 436 by males absent on the first (MOF), a histone acetyltransferase composed of two multiprotein complexes: the male-specific lethal complex (MSL) and nonspecific lethal complex (NSL) (Fig. 5). The latter acetylates LSD1 and inhibits, in this way, its binding to nucleosomes, thus contributing to the increase in the transcription of epithelial markers such as E-cadherin^116^.

LSD1 is also phosphorylated by the polo-like kinase 1 (PLK1) serine/threonine kinase that adds a phosphate group on serine 126. This modification promotes LSD1 release from chromatin during mitosis and maintains, in this manner, the correct balance between chromatin methylation and demethylation during the cell cycle^117^ (Fig. 5). LSD1 is also phosphorylated on serine 131 and 137 by casein kinase 2 (CK2α), binding under these modifications the ring finger protein 168 (RNF168 or E3 ubiquitin ligase) and p53-binding protein 1 (53BP1), favoring the activation of DNA damage repair^12^ (Fig. 5). Finally, LSD1 is subjected to PTMs that influence its stability: it can be, in fact, methylated on lysine 322 by suppressor of variegation 3–9 homolog 2 (SUV39H2), which inhibits the polyubiquitination and, consequently, degradation of LSD1 with a concomitant increase in its binding to the CoREST complex^118^ (Fig. 5).

### LSD1 as a regulator of protein stability through demethylase-independent activity

Recently, a new type of LSD1 activity has been highlighted: in several cases, it affects the stability of target proteins independent of its demethylating ability. For example, through its C-terminal domain, LSD1 interacts with the Cdc4 phosphodegron (CPD) motif of the F-box and WD repeat domain-containing 7 (FBXW7) protein, which acts as a substrate recognition subunit of the SKP-CUL1-F-box (SCF) E3 ubiquitin protein ligase complex that, in turn, mediates the ubiquitination and subsequent degradation of a series of important oncoproteins, such as Cyclin E, c-Jun, and c-Myc^119^. Upon binding to LSD1 through its CPD-binding site, FBXW7 loses affinity for its bonafide substrates and triggers its own ubiquitination and degradation^120^. In another example, the AOL domain of LSD1 interacts with the N-terminal region of the p62 protein, also known as sequestosome 1 (SQSTM1), that is a key component of the multiprotein complex that promotes autophagy^121^: LSD1 binding to p62 promotes its ubiquitination with proteasomal degradation and the subsequent inhibition of autophagy^122^. In contrast, the interaction of LSD1 with the ERRα orphan receptor results in stabilization of receptor turnover^123^.

## NeuroLSD1 (nLSD1) splicing variants

In addition to the role that LSD1 plays in stem cell renewal, cell proliferation and differentiation and tumor progression^124,125^, its function in the nervous system has been recently expanded with the discovery of a new neuron-specific alternative splicing variant named neuroLSD1^126^. This isoform does not substitute the other ubiquitously expressed (uLSD1) form but accounts for approximately 40–60% of the total protein present in the nervous system and, interestingly, shows an area-specific distribution, suggesting a fine-tuning mechanism that orchestrates the specific expression frame of both proteins^127^. nLSD1 differs from its sister variant in that in its AOL domain an extra tetrapeptide (K-V-T-D) is encoded by exon 8a, which is 12 nucleotides long and appears to be member of a “microexons” family consisting of a class of 3–15-nucleotide exons, that is essentially neuro-specific and adds variability to certain protein–protein interactions directly involved in the control of neuroplasticity and behavior^128,129^. More interestingly, this extra-tetrapeptide contains a threonine that can be phosphorylated, adding a further level of complexity to these protein–protein interactions. nLSD1 is unable to assemble with the CoREST complex and is then deprived of H3K4me1/2 activity^127^; moreover, it shares a common set of targets with uLSD1 but plays an opposite regulatory role. In fact, nLSD1 promotes transcription of genes repressed by the sister demethylase, which stimulates neuronal differentiation and acquisition of behaviors based on memory processes and emotions^130,131^. Notably, nLSD1 exhibits a novel methyl target represented by lysine 20 in histone H4, through which it regulates the expression of genes fundamental for cognitive functions such as learning and memory^130^.

The ratio of nLSD1 relative to uLSD1 is positively regulated by the master splicing regulators nSR100 and NOVA1 and negatively controlled by specific *cis*-acting sequences that prevent default expression of nLSD1 outside the nervous system^132,133^, making it conceivable that defects in splicing control may affect neurodevelopmental homeostasis^134,135^.

Interestingly, brain disorders implicating both LSD1 variants are being discovered: mutations in the catalytic domain shared by nLSD1 and uLSD1 that impair either protein stability or demethylase activity have been isolated^136,137^. In addition, a link between the excitability of the hippocampal circuitry and the level of nLSD1 has been highlighted in experimental models that mimic Rett syndrome (RTT) in which the methyl-CpG binding protein 2 (MeCP2) gene was knocked out^138^.

Finally, nLSD1 is not the unique variant highlighted in mammary neurons, as it is accompanied by two sister splice variants in which exon 2a is included, alone or in combination with exon 8a^127^. Interestingly, the LSD1-2a isoform is widely expressed in tissues, with the role of neurospecificity played by isoforms that include also the extra 8a exon^127^.

## Targeting LSD1 to fight against cancer

Given the key role played by LSD1 in carcinogenesis and because the multiple ways it interferes with a plethora of signaling pathways, targeting demethylase is emerging as a favorable option to treat cancer patients. Pharmacological inhibition of LSD1 with small molecules was shown to suppress cancer cell differentiation, proliferation, invasion, and migration, characterizing it as a novel therapeutic antitumor target^139^. In light of its biological importance, several LSD1 inhibitors have been developed, including natural products, peptides, and synthetic compounds that are currently undergoing clinical assessment for anticancer efficacy, especially in small lung cancer cells and acute myeloid leukemia. New and selective irreversible LSD1 inhibitors have been obtained from modifications on the phenyl ring and on the amino group of the monoamino oxidase (MAO) inhibitor tranylcypromine (TCP)^140,141^. TCP inhibits LSD1 in TCP-FAD adducts that establish van der Waals hydrophobic interactions with several LSD1 residues, changing the LSD1 conformation^142^.

Recently, several combinatorial therapies have been tested in which an LSD1 inhibitor has been added to different compounds. Below, we report some examples: the inhibition of LSD1 with TCP reactivates the all-trans retinoic acid (ATRA) differentiation pathway in nonacute promyelocytic leukemia (APL) and AML (in which ATRA alone is ineffective^143^) by inducing myeloid differentiation, suggesting the concomitant use of LSD1 inhibitor/ATRA. Mouse models confirm the synergistic effects of combined therapies with LSD1 inhibitors^144^. Currently, combinatorial therapies of LSD inhibitors (TCP, INCB059872, IMG-7289, and CC-90011) with chemotherapy (ATRA, cytarabine, azacitidine, cisplatin, and etoposide), monoclonal antibody (pembrolizumab), the inhibitor of indoleamine 2,3-dioxygenase-1 (IDO1) (epacadostat), histone deacetylase inhibitors (HDAC inhibitors)^145,146^, and the NEDD8-activating enzyme (NAE) inhibitor (pevonedistat)^147^ are under investigation for use in cancer therapy. Many other such protocols have been established where different drug associations show promising results for the treatment of several cancers, the exhaustive description of which is beyond the scope of this review^148^.

Finally, different groups have demonstrated that LSD1 scaffold activity, dynamically involved in carcinogenesis^149–152^, may be critical for unsuccessful therapies based on its catalytic inhibition in some cancers^152,153^. In particular, in AML cells resistant to LSD1 catalytic inhibition, the protein primarily represents a scaffold that recruits the CoREST complex to inhibit cell differentiation; in fact, inhibitors of LSD1 demethylase-independent activity induce the dissociation of the complex and responses to differentiation stimuli (such as RA)^150^. Moreover, LSD1 binds and destabilizes the tumor suppressor FBXW7, which by itself promotes the degradation of many oncoproteins independent of its demethylase activity^120^.

Given the multiple interactions of LSD1, these results are clinically relevant in all cases of cancer resistance to a single chemotherapy, further supporting the use of LSD1 inhibitors in combinatorial therapies.

## Concluding remarks

LSD1 was the first histone demethylase identified, and its presence on chromatin is crucial for the control of its plasticity through the regulation of histone methylation. Although in most cases excessive LSD1 activity is associated with the upregulation of cell growth and suppression of cell cycle regulatory proteins, it inhibits breast cancer metastasis in vivo^42^, suggesting that its behavior is highly context-dependent. Moreover, LSD1 shows a complex protein structure with a variety of noncatalytic domains and a lobed structure with a protruding hook that may account for a plethora of protein/protein interactions, that support the growing number of nonhistone proteins identified as its targets. To make matters more intriguing, noncatalytic interactions have been recently highlighted as relevant in controlling the progression and metastasis of a variety of cancers. These recent observations have paved the way for the discovery of many drugs used in cancer therapies based either on LSD1 demethylation or its noncatalytic activity, which involves its effect on protein half-life. Moreover, since several pivotal regulators of the cell cycle have been identified among its targets, this noncanonical activity of LSD1 is becoming increasingly relevant every day. In summary, LSD1 appears to be a multifaceted protein that is able to perform multiple tasks depending on its different domains. Therefore, it usually behaves as a double-edged demethylase that, when uncontrolled, displays very dangerous effects on cellular life. In fact, it is fine-tuned in very specialized circuitries that affect the meticulous control of gene expression and protein activation or degradation (i.e., the timely regulation of gene expression in development or in neuronal interactions to establish memory-based behavior).

Based on this evidence, the aim of anticancer therapies is merely to reestablish the original role of LSD1 as a fine-tuned epigenetic regulator. Of course, from this point of view, many questions must be addressed: for example, how do the noncatalytic domains of LSD1 interact with the catalytic pocket and how is the related cross talk regulated? Moreover, there is a high need for specific drugs that affect novel LSD1 features without any affecting others. However, despite its multifaceted effect on the cell cycle (or perhaps because of it), LSD1 appears to play a central role in the control of physiological and pathological cellular functions, that makes it an ideal target for new therapeutic strategies in the epic fight against cancer.
